# Enhancing Humidity Sensing with Functionalized Perylene-Coated Dispense Printed Electrodes: A Comparative Study

**DOI:** 10.1021/acsaelm.5c00360

**Published:** 2025-06-27

**Authors:** Sahira Vasquez, Samuel Morales-Cámara, Carmen Moraila, Yann Houeix, Isabel Blasco Pascual, José F. Salmerón, Antonio Rodríguez-Diéguez, Sara Rojas, Niko Münzenrieder, Luisa Petti, Paolo Lugli, Almudena Rivadeneyra

**Affiliations:** † Faculty of Engineering, Free University of Bolzano-Bozen, via Bruno Buozzi 1, 39100 Bolzano, Italy; ‡ Department of Inorganic Chemistry, Faculty of Science, 16741 University of Granada, 18071 Granada, Spain; § Department Electronics and Computer Technology, 16741 University of Granada, 18071 Granada, Spain; ∥ Department of Analytical Chemistry, 16741 University of Granada, 18071 Granada, Spain

**Keywords:** Perylene diimides, dispense printing, material synthesis, ligands, relative humidity

## Abstract

Emerging sensing materials are central to improving the functionality and integration of electronic devices. In this study, we report the synthesis of a custom perylene-based organic ligand, *N*,*N*′-di­(phenyl-3,5-dicarboxylic acid)-perylene-3,4:9,10-tetracarboxylic acid diimide (PY), which exhibits a strong response to relative humidity (RH). Its sensing performance was systematically compared with that of a commercially available perylene derivative, *N*,*N*′-bis­(4-methoxy-benzyl)­perylene-3,4:9,10-bis­(dicarboximide) (PBI). Both materials were deposited onto polyimide substrates with silver-based dispense-printed interdigitated electrodes to fabricate impedimetric sensors. The PY-based sensor demonstrated a high sensitivity of −5289 Ω/% RH at 1 kHz within a 30–90% RH range while exhibiting minimal temperature dependence. In contrast, the PBI-based sensor showed a lower humidity sensitivity of −452 Ω/% RH and a negligible temperature response. This study highlights the potential of functionalized perylene derivatives for developing high performance humidity sensors with minimal thermal interference, eliminating the need for temperature compensation and enabling integration into low power electronic systems. These findings provide valuable insights into molecular design strategies for next-generation environmental monitoring and flexible electronic applications.

## Graphical abstract

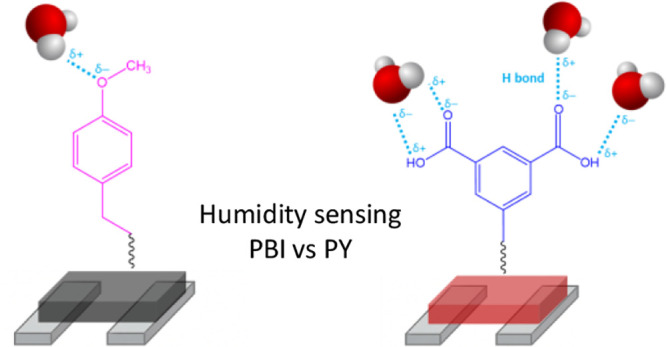

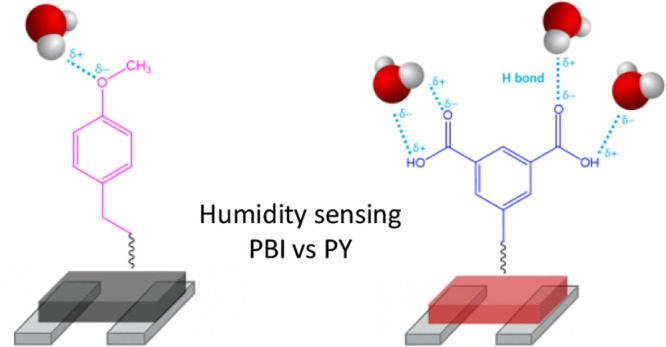

## Introduction

The Internet of Things (IoT) has transformed the landscape of modern living, enabling seamless data connectivity among objects and their environment.^1^ Among its numerous applications, environmental data collection has emerged as a critical achievement, with humidity being one of the most essential parameters to monitor.^2^ Accurate temperature and humidity measurements in IoT systems enable informed decision-making, efficiency improvements, and enhanced well-being across various fields, including environmental monitoring,^3,4^ quality control,^5,6^ energy management,^7^ smart agriculture,^8,9^ cold chain logistics,^10,11^ medical applications,^12^ and indoor environmental control.^13^

As the demand for precise and unobtrusive environmental sensing in IoT systems continues to grow, the electronics industry is increasingly aiming for ubiquitous computation, an approach in which flexible thin-film devices play a pivotal role.^14–16^ Among the diverse range of sensors required for this vision, flexible humidity sensors are especially crucial. However, despite significant progress in the field, many existing flexible humidity sensors still suffer from critical limitations that hinder their adoption in demanding applications.

A major limitation of current flexible humidity sensors is their susceptibility to temperature variations, which introduce measurement inaccuracies and require additional compensation techniques. Most commercially available sensors rely on capacitive or resistive transduction mechanisms, where humidity-induced changes in dielectric properties or resistance are also affected by temperature fluctuations, resulting in erroneous readings.^14,17^ This issue is particularly critical in high precision applications, such as environmental monitoring and medical diagnostics, where even small errors can significantly impact decision making. In addition to temperature sensitivity, printed humidity sensors often exhibit slow response and recovery times due to the slow diffusion of water molecules within the sensing layer. This limitation undermines their suitability for real-time monitoring applications, where the rapid detection of humidity fluctuations is essential. Hysteresis is another persistent challenge, sensor outputs frequently depend on previous humidity exposure, compromising repeatability and long-term reliability.^18,19^ Furthermore, there exists a trade-off between sensitivity and operational range. Many printed sensors fail to maintain a high sensitivity across a wide humidity spectrum, limiting their performance in environments with variable humidity levels. Long-term stability also remains a concern as degradation caused by material instability or contamination often leads to performance drift, requiring frequent recalibration or replacement and increasing operational costs. Despite increasing interest in solution-processable materials and low-cost fabrication strategies, the field still lacks printed humidity sensors that can effectively overcome these challenges in a scalable, reliable, and sustainable manner.

Given these limitations, there is an increasing demand for printed humidity sensors that combine high sensitivity, rapid response times, minimal hysteresis, and stable performance under varying environmental conditions. In this context, various humidity-sensitive materials, such as metal oxides, polymers, carbon-based materials, and organic semiconductors, have attracted considerable attention in recent years due to their unique advantages. These include solution processability, cost-effective fabrication, room-temperature operation, straightforward integration, and scalable synthetic approaches.^20,21^

Among emerging materials, functionalized perylene-based compounds offer a particularly promising avenue for addressing current limitations in humidity sensing.^22^ Although perylene diimides (PDIs) have been extensively studied in the context of organic electronics, their potential for humidity sensing remains largely unexplored, making their exploration a valuable step toward broadening their application scope. By tailoring the chemical functionalities of PDIs, it is possible to enhance their interaction with water molecules while simultaneously minimizing the temperature-related variability.

Originally introduced as textile dyes,^23^ PDIs are characterized by strong π–π stacking interactions, excellent thermal and photostability under ambient conditions, and remarkable resistance to oxidative degradation. Their electron-withdrawing imide groups facilitate stable chemical and electrochemical reductions, reinforcing their status as robust n-type semiconductors.^24^ Furthermore, PDIs are highly amenable to structural modifications, such as the introduction of alkyl or aryl substituents at the imide nitrogen atoms or functionalization at the bay positions of the aromatic core. These modifications allow for the fine-tuning of their electronic and optical properties, enabling customization for specific sensing or electronic applications.^23–25^

To investigate these opportunities, a custom-synthesized *N*,*N*′-di­(phenyl-3,5-dicarboxylic acid)-perylene-3,4:9,10-tetracarboxylic acid diimide (PY), functionalized with carboxylic acid groups, was specifically engineered to enhance humidity sensitivity and reduce temperature dependence, making it a compelling alternative to commercial solutions. To comprehensively compare the sensing properties of this material, *N*,*N*′-bis­(4-methoxybenzyl)­perylene-3,4:9,10-bis­(dicarboximide) (PBI) was selected as a benchmark film due to its established use in sensing, allowing a direct comparison with the custom-designed PY derivative. It features a conjugated perylene core and functional moieties capable of interacting with environmental stimuli. While this specific compound has not been extensively explored as a humidity-sensing layer, its structural similarity to other PBI derivatives that have demonstrated effective sensing capabilities provides a strong foundation for its investigation in this context.^26,27^ The incorporation of methoxybenzyl substituents introduces hydrophilic characteristics that can influence the interaction of the material with water molecules, making it a promising candidate for humidity sensing.^28,29^ Additionally, its chemical stability and compatibility with flexible substrates further support its suitability for integration into low-power, flexible electronic sensing devices.

In this work, we report the fabrication of humidity-sensing layers with minimal temperature dependence using two PDI derivatives: the commercially available PBI and the custom-synthesized PY. Devices were fabricated by using printed silver (Ag) interdigitated electrodes (IDEs) coated with both PDI derivatives and characterized as impedimetric sensors. The PY based sensor demonstrated much higher sensitivity to humidity compared with the PBI based sensor. In terms of the temperature response, both sensors showed minimal or no significant sensitivity to temperature variations. These results demonstrate that functionalization at the N-position of PDIs significantly improves the sensor response and selectivity with respect to temperature. Furthermore, this functionalization eliminates the need for additional temperature sensors or compensation algorithms, reducing the system size, processing time, and overall cost. The proposed humidity sensors, characterized by their flexibility, low power consumption, and thermal stability, are well suited for the integration into IoT platforms. These features make them particularly attractive for applications such as smart agriculture, where localized microclimate monitoring is essential for precision irrigation and disease prevention. Additionally, their conformability enables their use in wearable systems for personal comfort and health tracking as well as in cold-chain logistics, where fast and reliable humidity detection is critical for ensuring the quality and safety of perishable goods. These use cases highlight the practical relevance and technological potential of our sensors in distributed sensing networks.

## Materials and Methods

All reactants were commercially obtained and used without further modification. *N*,*N*′-bis­(4-methoxybenzyl)­perylene-3,4:9,10-bis­(dicarboximide) (PBI, 99%), perylene tetracarboxylic dianhydride (97%), imidazole (99%) and 5-aminoisophtalic acid (97%) were purchased from Sigma-Aldrich.

### General Instrumentation

Elemental analyses (EA) were carried out on a Thermo Scientific analyzer, Flashh 2000. ^1^H NMR spectra were acquired on a 300 MHz Varian Equipment using deuterated water as solvent. Fourier-transform infrared (FTIR) spectroscopy was carried out using a Bruker FTIR spectrometer (Bruker Optik GmbH, Germany) to confirm the presence of functional groups in the PBI and PY compounds. The morphological features of the deposited sensing material and printed electrodes were investigated using a light microscope (DM8000 M, Leica Microsystems CMS GmbH, Wetzlar, Germany). SEM images were obtained with an NVision40 FESEM from Carl Zeiss (Oberkochen, Germany) at an acceleration voltage of 5 kV. To evaluate the topography and uniformity of the deposited sensing layers, 3D optical profilometry images were acquired using a confocal laser scanning microscope (Keyence VK-X Series). Finally, the sensor response to humidity was tested in a humidity chamber (Weiss Technik, LabEvent L C/34/70/5) using an impedance analyzer (Keysight E4990A) with an attached probe (Keysight 42941A) from Agilent Technologies.

### Synthesis of *N*,*N*′-Di­(phenyl-3,5-dicarboxylic acid)-perylene-3,4:9,10-tetracar boxylic Acid Diimide (PY)

The synthesis of PY was performed according to the previously reported procedure.^30^ In brief, 2.00 g (5.10 mmol) of perylene tetracarboxylic dianhydride, 10 g (146.89 mmol) of imidazole, and 2.308 g (12.74 mmol) of 5-aminoisophtalic acid were mixed together and heated at 130 °C for 6 h under an argon atmosphere. After the mixture was cooled to room temperature, 150 mL of ethanol was added to the mixture and heated again at reflux temperature overnight. A precipitate was collected from the reaction mixture by cooling to room temperature and washed three times with 50 mL of ethanol, filtrated, and dried at room temperature to get deep red powder-like precipitation. Anal. Calcd for C_40_H_18_N_2_O_12_: C, 66.86; H, 2.52; N, 3.90%. Found: C, 66.78; H, 2.49; N, 3.95%. ^1^H NMR (300 MHz, DMSOd-6): Δ (ppm), 7.65 (s, 4H), 7.37 (s,2H), 7.02 (s,8H).

The chemical structures of the synthesized compound (PY) and the commercial one (PBI) are presented in Figure 1a and b, respectively.

**Figure 1:**
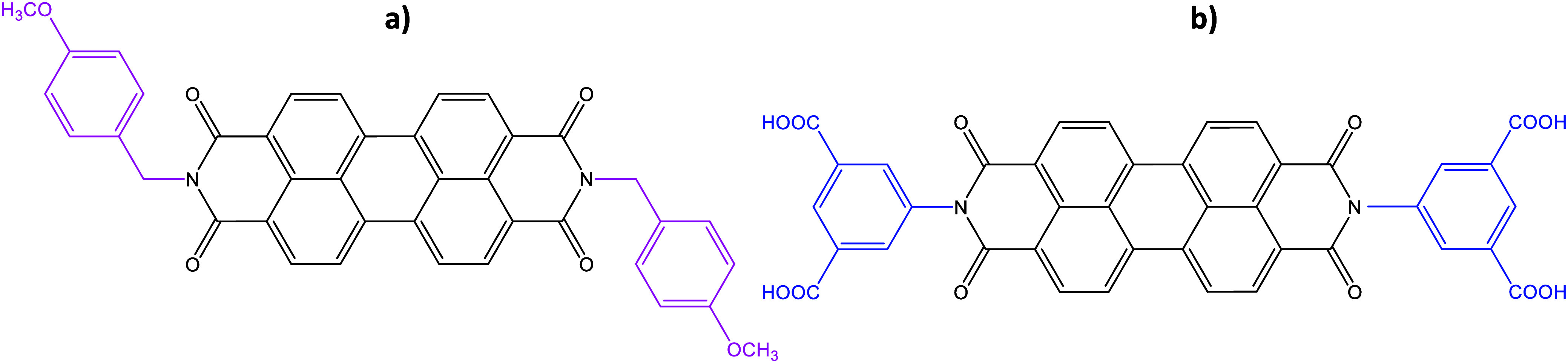
Chemical structure of the functionalized perylene derivatives used as sensing materials: (a) PBI is functionalized with methoxybenzyl groups, which are highlighted in pink. (b) PY is functionalized with carboxylic groups, which are highlighted in blue.

### Device Fabrication

The steps to fabricate the flexible dispense printed sensor are shown in Figure 2. A layout of 8 fingers, each 200 μm wide with a gap of 400 μm, was designed in Adobe Illustrator (Figure 2 a). The commercial dispense printer (Voltera, V-One 5000), shown in Figure 2b, was used to dispense highly conductive Ag paste (Voltera, Conductor) onto polyimide (PI) (Kapton HN, DupontTM, Wilmington, DE, USA). The PI substrate was flattened onto a printed circuit board (PCB) using droplets of water and secured with tape to eliminate any gaps between the substrate and the underlying surface, thereby ensuring uniform printing. For printing, the trim length was set to 150 mm, the antistringing distance to 0.1 mm, and the kick distance to 2 mm. This ensured smooth and consistent material deposition,^31^ while the Z-height was adjusted to 0.7 mm to improve adhesion and minimize line spreading. Finally, the electrodes were cured in an oven (Memmert GmbH) at 120 °C for 20 min.

**Figure 2:**
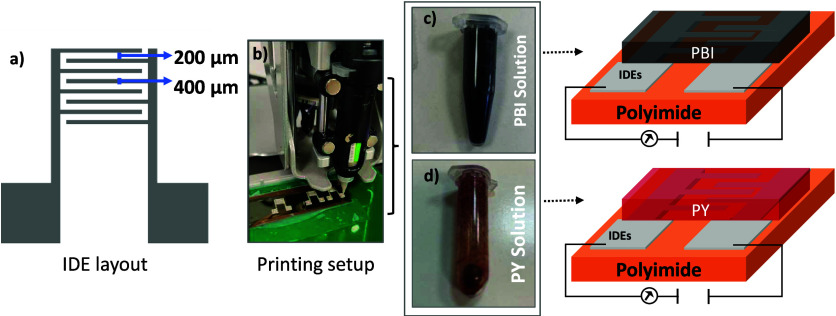
Fabrication and structure of the printed humidity sensors: (a) Schematic of the interdigitated electrode (IDE) layout with labeled dimensions. (b) Printing setup using dispense-printing for IDE fabrication on flexible polyimide substrates. (c) Photograph of the PBI solution. (d) Photograph of the PY solution. Right panels illustrate the device structure for both PBI and PY based sensors, with the sensing layers coated onto printed silver IDEs.

The sensing polymers PY and PBI were dissolved in distilled water with a weight content of 0.6 wt % and subsequently sonicated (Ultrasonic Cleaner, JP-008) until complete dissolution. The as-prepared solution was used as the humidity sensing layer. As previously shown in,^32^ deposition by means of drop casting does not significantly affect the sensor response to humidity when compared to large-scale manufacturing techniques such as spray coating. Thus, to enable a very quick, cost-effective, and easy method, we deposited 30 μL of the sensing layer in a controlled volume with a plastic volumetric pipet to cover the entire active area. After deposition, the solution was dried in the oven at 100 °C for 10 min to evaporate the water and adhere the solid material to the electrodes and substrate. Finally, to test the sensor toward humidity, the pads were contacted with conductive paint (Bare Conductive Ltd., UK) to a coaxial PBC connector and subsequently to an SMA plug to SMA plug RF adapter. Images of the as-fabricated sensors are presented in Figure S1.

### Device Characterization

The sensor’s impedance was evaluated under sequential humidity steps from 30% to 90% RH, then back down, with each humidity level maintained for 30 min at a fixed temperature of 30 °C. The phase and magnitude responses were extracted across a frequency range of 500 Hz to 10 MHz with an amplitude of 500 mV. Additional temperature measurements were performed using the same instrument, spanning a temperature range of 30 to 80 °C at a constant humidity level of 55% over the same frequency range. Each experiment was performed on three independently fabricated sensors per material, and all measurements were conducted in triplicate to ensure statistical reliability. The results are reported as mean values with standard deviations to illustrate the data variability.

## Results

### Physicochemical Characterization

^1^H NMR (300 MHz, DMSO-*d*_6_) analysis confirmed the successful synthesis of the PY ligand. As shown in Figure S2, the aromatic protons of the perylene core resonate at 7.02 ppm, whereas the isophthalic acid fragment gives signals at 7.37 ppm (2 H, H_C_) and 7.65 ppm (4 H, H_B_). Complete spectral data: ^1^H NMR (DMSO-*d*_6_:7.02 (s, 8H, Ar–H), 7.37 (s, 2H, Ar–H) and 7.65 (s, 4H, Ar–H) corresponding to *H*_*A*_, *H*_*C*_, and *H*_*B*_ protons, respectively. The integral ratio of H_A_:H_C_:H_B_ = 8:2:4 matches the theoretical value, and no resonances attributable to unreacted precursors are detected. Minor residual peaks correspond to ethanol and water from the preparation protocol as well as to the DMSO-*d*_6_ solvent.

FTRI spectroscopy was used to confirm the presence of functional groups in the PBI and PY compounds. As shown in Figure 3, both materials exhibit strong absorption bands associated with their shared perylene diimide core. Prominent bands appear in the ranges of 1705–1775 and 1660–1690 cm^–1^, corresponding to the asymmetric and symmetric C=O stretching vibrations of the imide groups, respectively. Additionally, characteristic aromatic C=C and C–H stretching bands are observed near 1600 and 2900 cm^–1^, respectively. PBI shows additional peaks around 1250 and 1025 cm^–1^, attributed to C–O–C and C–O stretching vibrations from methoxy substituents. In contrast, PY displays a peak at 1700 cm^–1^ assigned to the C=O stretching of carboxylic acid groups, along with a broad band in the 2500–3000 cm^–1^ region associated with O–H stretching. The wagging vibration of the carboxylic O–H group is also detected at 651 cm^–1^. These findings confirm the targeted functional groups in the perylene-based structures.

**Figure 3:**
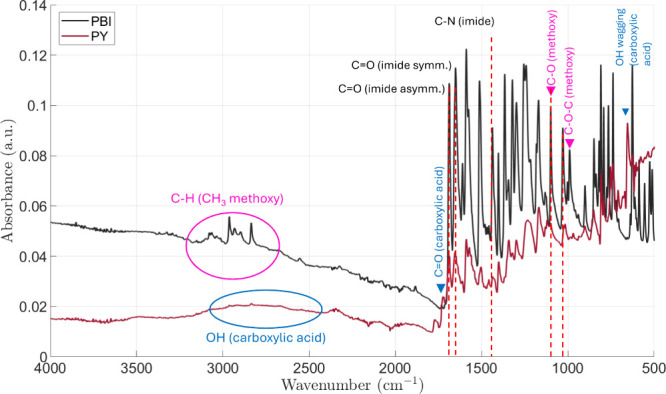
FTIR spectra of PBI and PY compounds.

To evaluate the dimensional consistency and quality of the printed silver IDEs, optical microscopy images were acquired before the deposition of the active sensing layers. As illustrated in Figure S3, the width of the printed silver lines was found to increase by approximately 22%, compared to the design specifications, while the spacing between electrodes decreased by about 13%. These variations are consistent with previous studies on dispense-printed electrodes using similar setups.^33^ Despite these deviations from nominal values, the printed features demonstrated uniformity across the entire substrate, ensuring a reproducible sensor performance. The data confirm that while a nominal gap of 200 μm may result in a slightly narrower printed gap, the overall effect on device behavior is negligible due to the reproducible patterning. Furthermore, Figure 4 shows optical microscope images of the drop-cast PBI and PY sensing layers on the printed electrodes. These images reveal a uniform coverage of both materials with distinct morphological differences: PBI films exhibit a more granular and compact structure, whereas PY films show spherical features with a more heterogeneous distribution. To investigate the quality of the coating process further, 3D optical profilometry was performed on the PBI and PY coated sensors. As Figure 5 shows, both films exhibit uniform coverage across the IDEs area. Notably, the PY layer has a greater overall thickness and more pronounced topography than the PBI coating, which could affect the water vapor adsorption dynamics. These profiles confirm the reliable deposition of the two functional materials and their morphological distinction.

**Figure 4:**
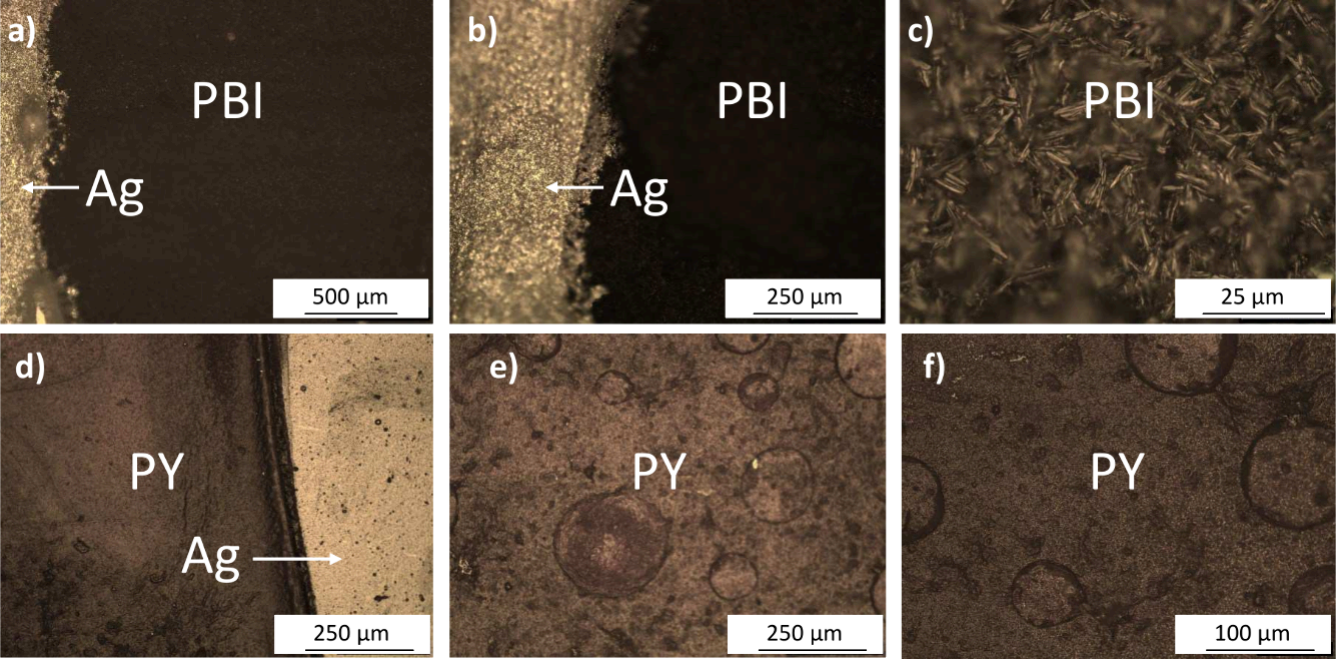
Optical microscope images of the printed silver interdigitated electrodes (IDEs) and drop-cast sensing layers. (a–c) PBI films showing a compact granular structure and good adhesion to the printed silver IDEs. (d–f) PY films exhibiting spherical aggregates and heterogeneous surface coverage.

**Figure 5:**
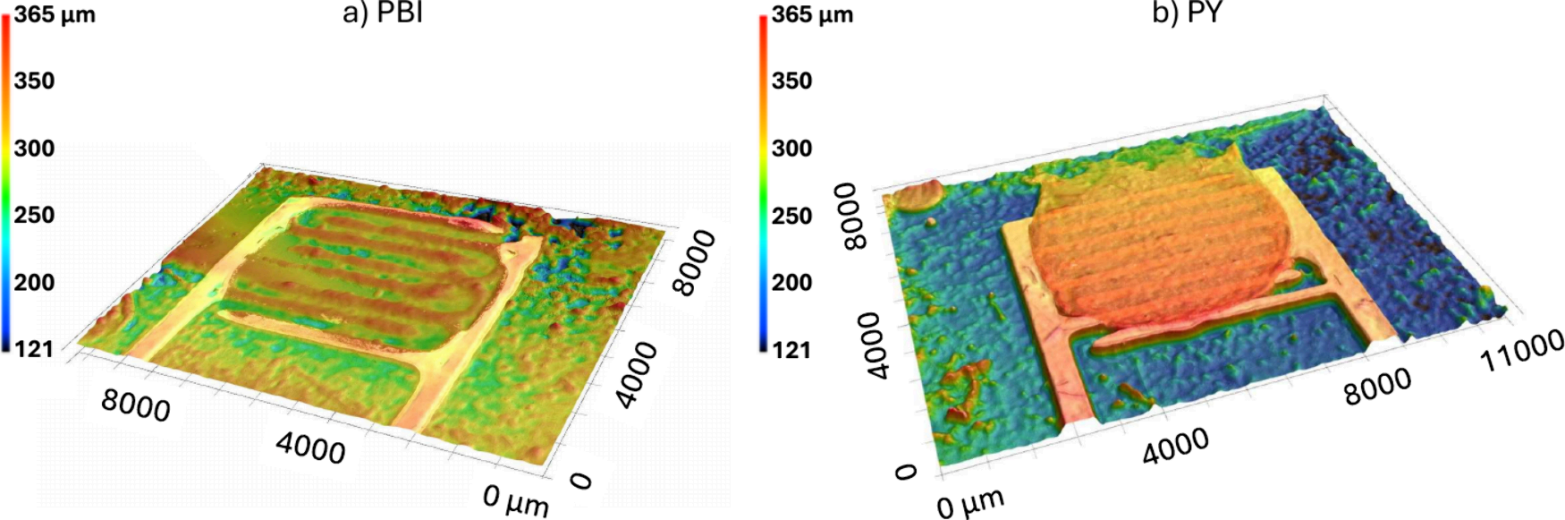
3D optical images of the sensing area coated with (a) PBI and (b) PY films on Ag-based interdigitated electrodes (IDEs). The color scale indicates surface height variations, confirming the topographical differences between the two functionalized layers.

Building upon the optical and profilometry analysis, Figure 6 presents SEM images that offer a more detailed view of the microstructural differences between the PBI (a-c) and PY­(d-f) sensing layers. The PY film exhibits spherical aggregates uniformly distributed across the substrate (Figure 6a). Upon closer examination (Figure 6b), these aggregates reveal a fibrous surface texture, likely arising from strong π–π stacking interactions that promote anisotropic molecular assembly. The cross-sectional view (Figure 6c) shows a dense central core surrounded by outwardly protruding fibrils, indicating morphological heterogeneity that may support a diverse array of water adsorption sites. In contrast, the PBI film presents a granular surface texture at lower magnification (Figure 6d), which transitions into densely packed, needle-like crystalline domains at higher magnifications (Figure 6e and f). These rod-shaped features, likely driven by the methoxybenzyl side chains, form an interconnected porous framework that can facilitate water vapor penetration and diffusion throughout the sensing layer. The marked differences in film morphology between PY and PBI suggest distinct humidity adsorption mechanisms and transport properties, which are expected to significantly impact their respective sensing performances, as discussed in the following section.

**Figure 6:**
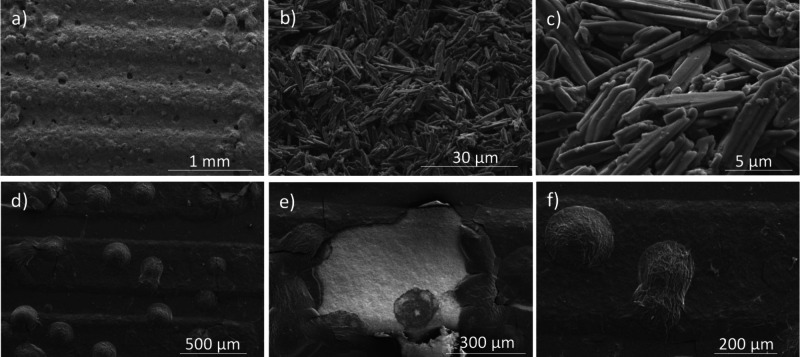
SEM images of PBI (a), (b) and (c) and PY (d), (e), and (f) at different magnifications.

### Response toward Humidity

The organic dielectric materials (PBI and PY) deposited between the interdigitated fingers form a capacitor, where the interaction with water vapor molecules alters the capacitance, thus changing the impedance response. Figure 7 summarizes the frequency-dependent impedance of the two sensor during the upward humidity sweep (30–90% RH, mean of three devices). Panels (a) and (b) display the phase angle and impedance magnitude of the PBI sensor, while panels (c) and (d) present the corresponding data for the PY sensor. Each colored trace represents a discrete RH set-point, and the spectra span 500 Hz–10 MHz. Figure 8 shows the same four plots for the downward sweep (90 → 30% RH).

**Figure 7:**
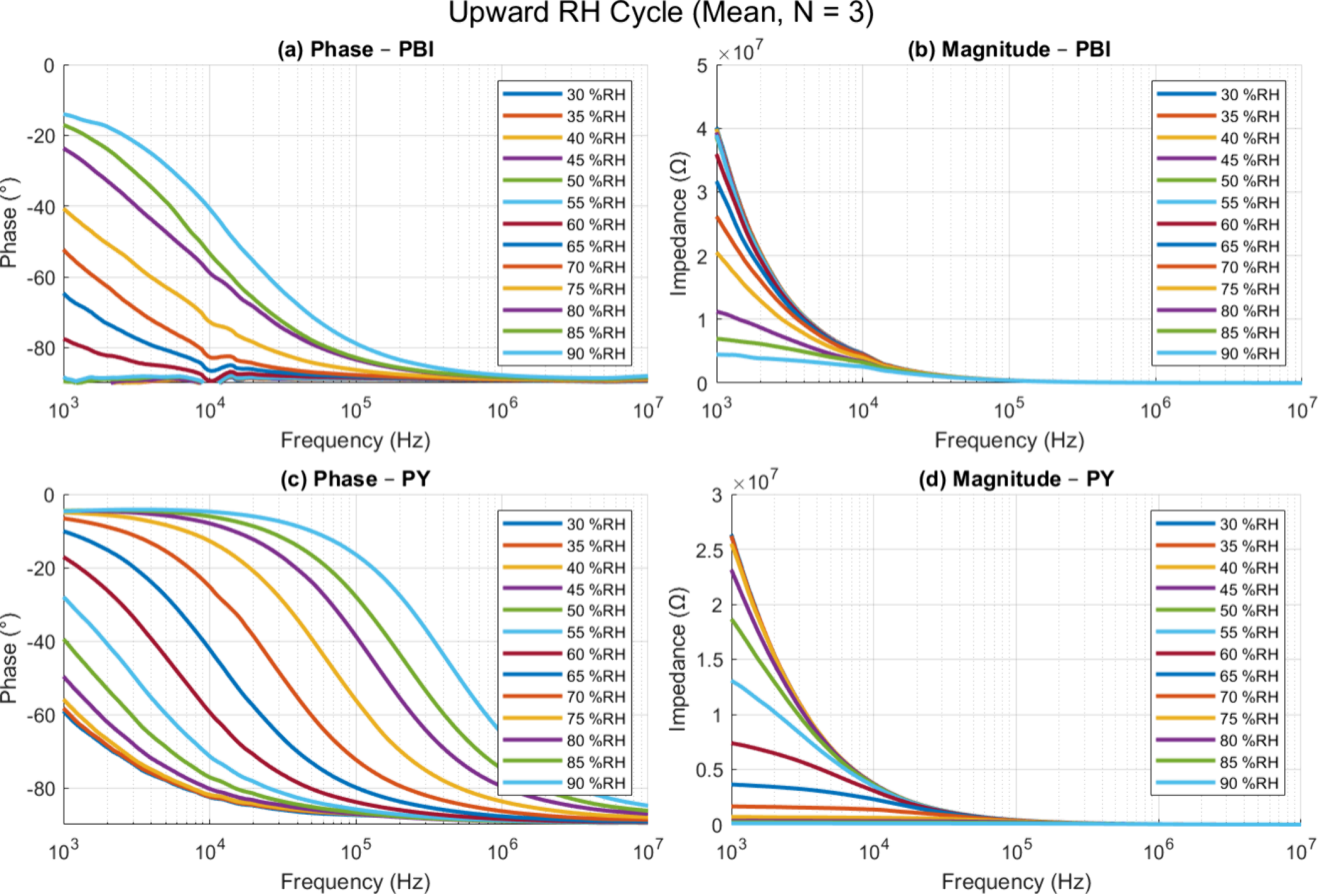
Frequency-dependent impedance spectroscopy measurements of printed humidity sensors based on PBI and PY materials under varying relative humidity (RH) levels from 30% to 90% at 30 °C. (a, b) Phase and magnitude responses of the PBI sensor. (c, d) Phase and magnitude responses of PY based sensor. Data represent the mean values obtained from three independent sensor samples (*N* = 3) for each material.

**Figure 8:**
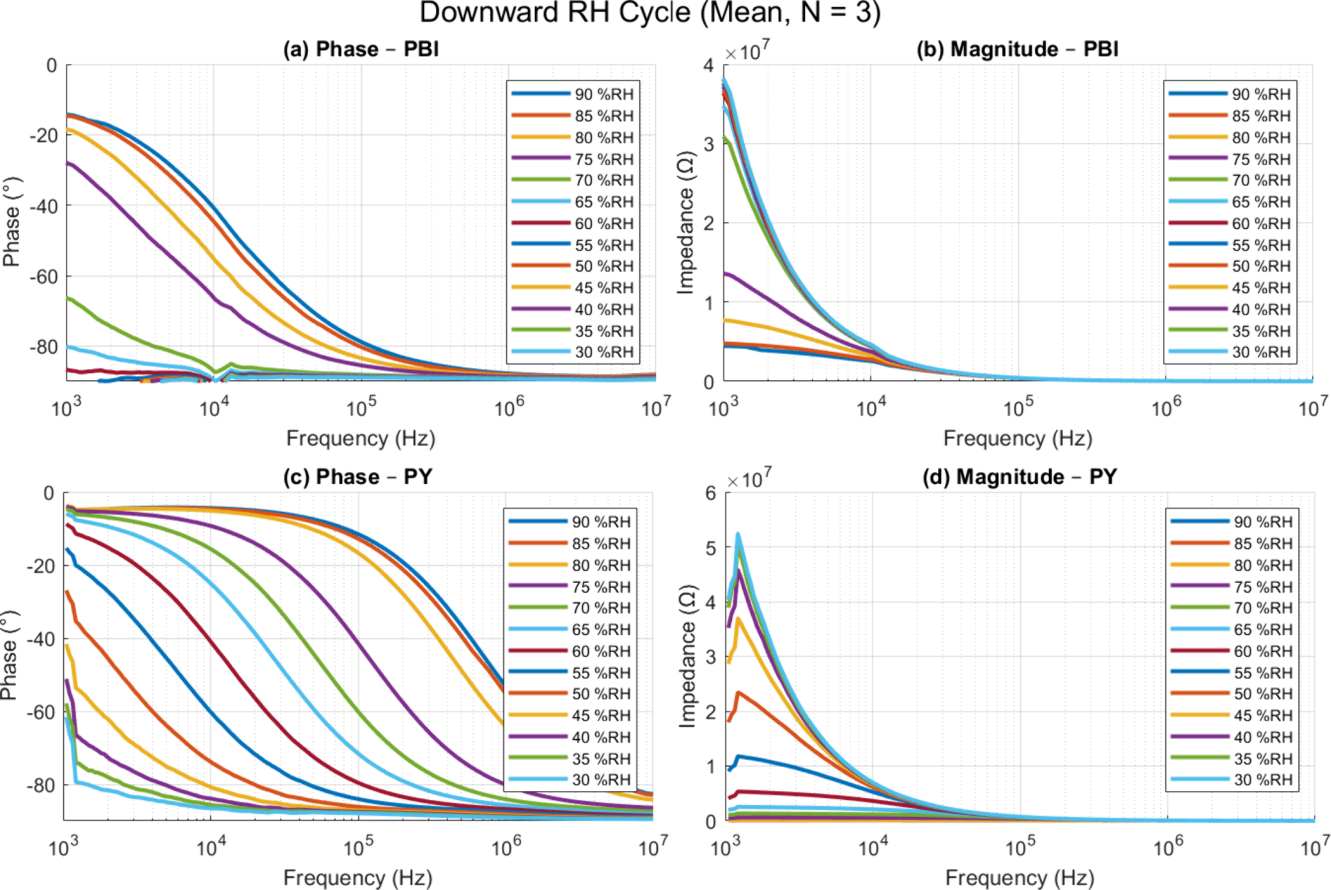
Frequency-dependent impedance spectroscopy measurements of printed humidity sensors based on PBI and PY materials under varying relative humidity (RH) levels from 90% to 30% at 30 °C. (a, b) Phase and magnitude responses of the PBI sensor. (c, d) Phase and magnitude responses of the PY based sensor. Data represent the mean values obtained from three independent sensor samples (*N* = 3) for each material.

In both materials, increasing humidity causes a drop in the impedance magnitude and a phase angle that shifts toward 0°, behavior consistent with increasing ionic conductivity and permittivity in the sensing layers. The PY sensors (panels (c) and (d)) show a smooth, nearly monotonic phase evolution, especially below ∼10 kHz, implying uniform and reversible water uptake. By contrast, the PBI devices (panels (a) and (b)) show a broader spread in phase at intermediate RH levels (50–70% RH), a response that is potentially linked to its more heterogeneous texture revealed by SEM and optical profilometry. Figures S4 and S5 present the complete spectra for PBI and PY, respectively; each curve is the mean of three independent measurements, and the shaded regions indicate the SD.

Figure 9 shows the calibration curves acquired at 1 kHz. This single representative frequency is shown to maintain the figure clarity. Both upward (up) and downward (down) sweeps are shown to visualize the hysteresis effects.

**Figure 9:**
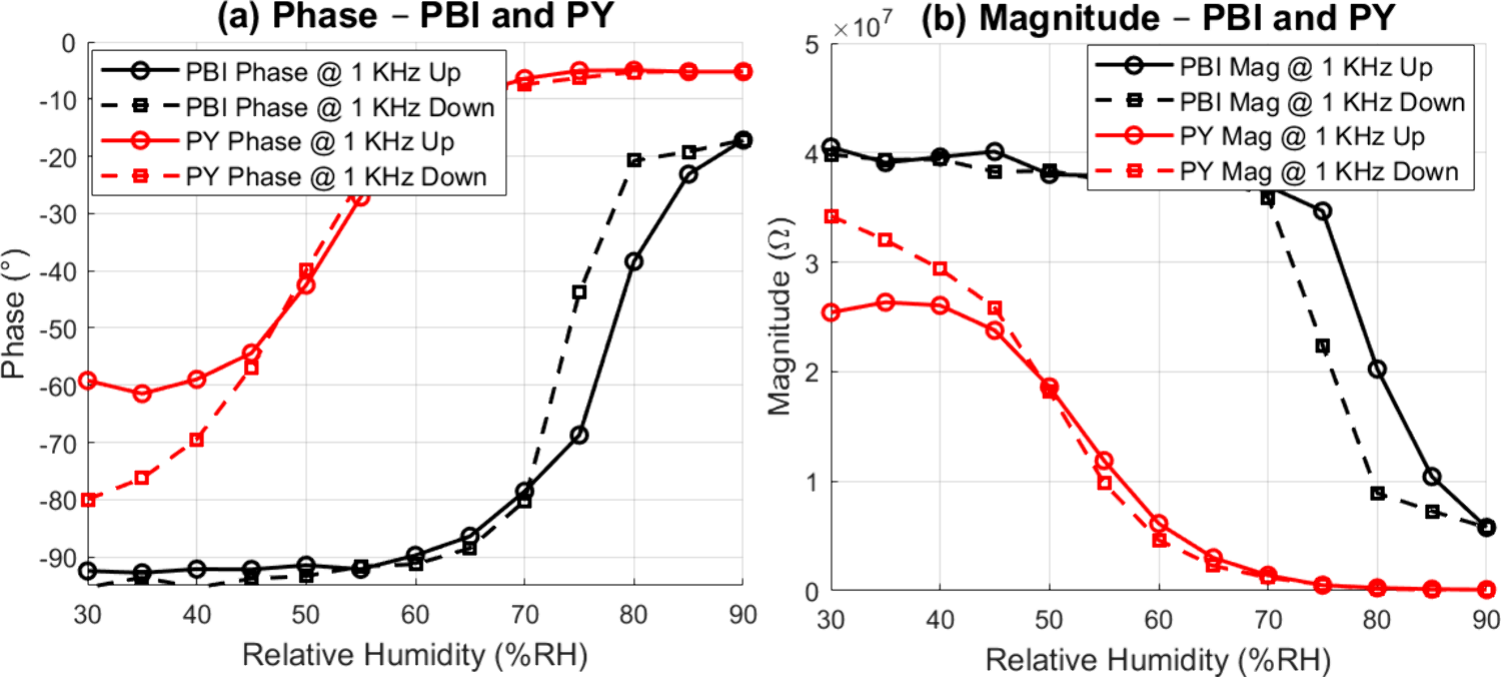
Calibration curves of the printed PBI and PY humidity sensors at 1 kHz. (a) Phase response versus relative humidity shows clear differences in sensitivity between the two materials, with PY demonstrating greater phase shifts over the measured range. (b) Impedance magnitude response indicates consistent humidity dependence, with PY again showing higher sensitivity than PBI. Measurements were collected during both increasing (“up”) and decreasing (“down”) humidity cycles to assess hysteresis.

Up to ∼55  % RH, the phase angle of the PBI coated sensor is nearly constant, attributed to a dominantly capacitive response with limited water uptake. Beyond this threshold the phase starts to shift toward 0°, while the impedance decreases, giving a sigmoidal transfer curve. The sharp transition is consistent with the methoxy-substituted PBI backbone, which hinders adsorption until a critical humidity is reached; subsequent clustering of water molecules facilitates proton transport and lowers impedance.^34^ In contrast, the PY sensor responds more linearly across the entire 30–90% RH window. Both phase and magnitude change gradually and reproducibly, reflecting the higher hydrophilicity imparted by multiple carboxylic-acid groups that engage in hydrogen bonding.^35^

Both materials exhibit hysteresis but with different signatures. For PBI, the entire phase trajectory is displaced during the down-sweep, producing an average hysteresis of ∼9% in the phase trace. For PY, hysteresis manifests mainly as a baseline offset: at the starting 30% RH the down-sweep phase is about −80°, whereas the up-sweep starts near −60°. Above ∼55% RH the two PY curves nearly coincide; therefore, the integrated hysteresis over the full cycle is limited to ∼4%. This baseline shift implies that a small amount of water is retained in the PY layer after exposure to high humidity yet desorption is sufficiently rapid that the response is essentially reversible once RH exceeds the sorption threshold.

A quantitative summary of the sensitivities for both sensors at different frequencies is presented in Table 1. In summary, PY offers better sensitivity and reversibility, while PBI delivers a steeper, switch like response once its adsorption threshold is crossed, potentially suiting applications where a sharp humidity trigger is required.

**Table 1:** Sensitivity of Sensor in Linear Response and Range of RH of Linear Impedance Response

RH range (%)	Frequency	PY (deg)	PBI (deg)	PY (Ω)	PBI (Ω)
30–90	1 kHz	1.29	0.15	–5289	–452
30–90	10 kHz	0.34	0.024	–119	–23
30–90	100 kHz	0.075	0.026	–3433	–1693

The origin of these behaviors is clarified in Figure 10, which schematically depicts how water molecules interact with the chemically functionalized perylene derivatives. Water adsorption alters the local dielectric environment, resulting in changes to both permittivity and ionic conductivity. These changes affect the impedance spectra, specifically the phase angle and magnitude, recorded across the IDEs. The nature and strength of the interactions between water and the material are governed by the chemical functionalities present in each compound. The PY derivative functionalized with carboxylic acid groups enables strong hydrogen bonding interactions with water molecules, acting as both a donor and an acceptor. This dual interaction capability enhances water uptake, increasing the effective dielectric constant and facilitating ion transport within the sensing film. Consequently, PY based sensors exhibit more pronounced impedance shifts and a broader dynamic response to humidity changes. In contrast, the PBI derivative bearing methoxy substituents interacts more weakly with water molecules. These groups can only function as hydrogen bond acceptors, which limits water adsorption and reduces the degree of dielectric modulation under varying RH levels. Consequently, PBI sensors are less sensitive than PY based sensors, especially at low humidity concentrations. These interaction differences also affect the sensors’ long-term performance and stability. The stronger and more uniform water binding in PY may contribute to better reproducibility and reduced signal drift. In contrast, the heterogeneous adsorption in PBI could result in greater variability. Together, these insights support the rationale approach of selecting functional groups for designing humidity sensors and help explain the distinct impedance behavior observed between the two materials.

**Figure 10:**
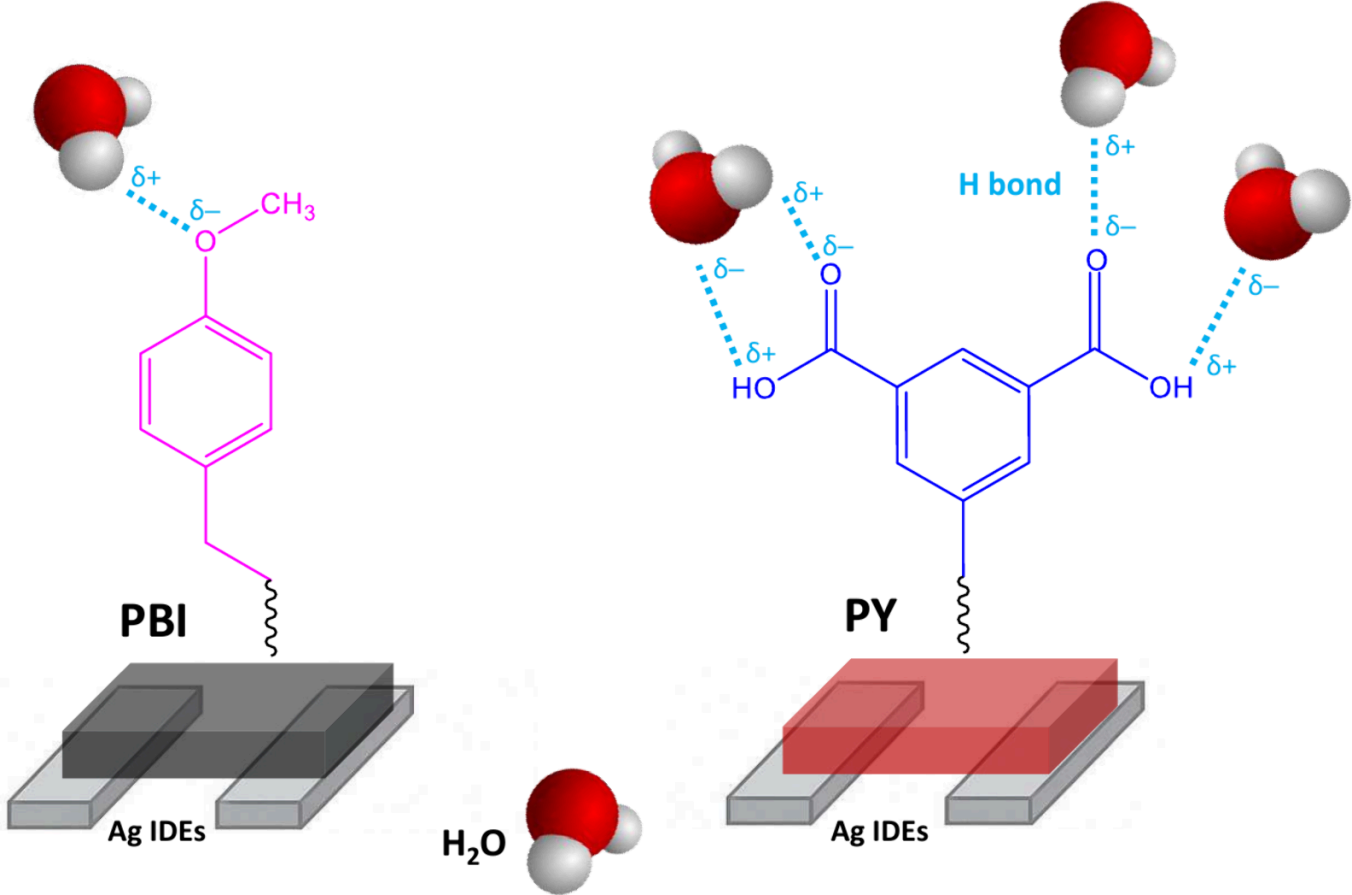
Schematic illustration of the humidity sensing mechanism for the functionalized perylene diimide derivatives: PBI with methoxybenzyl groups (left) and PY with carboxylic acid groups (right).

To represent the impedance behavior of the printed impedimetric humidity sensors, an equivalent circuit model consisting of a resistor in parallel with a capacitor was adopted, as shown in Figure 11. This configuration effectively captures both the resistive and capacitive responses of the sensing film. The parallel resistance accounts for the resistive nature of PBI and PY, while the capacitive element reflects the inherent capacitance of the IDEs and the effect of the permittivity of the top layer. At low relative humidity, the impedance response is predominantly capacitive. As humidity increases, the resistive component becomes more significant as a result of decreasing resistance. In the case of the PBI sensor, a sharp transition from capacitive to resistive behavior is observed around 55% RH, whereas the PY sensor shows a smoother transition. The capacitance is similar for both sensors, as it is inherent to the electrode design. Variations in capacitance with humidity are attributed to changes in permittivity caused by the absorption of water molecules. Using phase and magnitude data obtained from electrochemical impedance spectroscopy, we applied this equivalent circuit model using particle swarm optimization (PSO) for all the sensors. The fitted results are summarized in Table 2, obtaining an average R^2^ for magnitude over 0.997 and for phase 0.835 for all 6 sensors and across all RH range.

**Figure 11:**
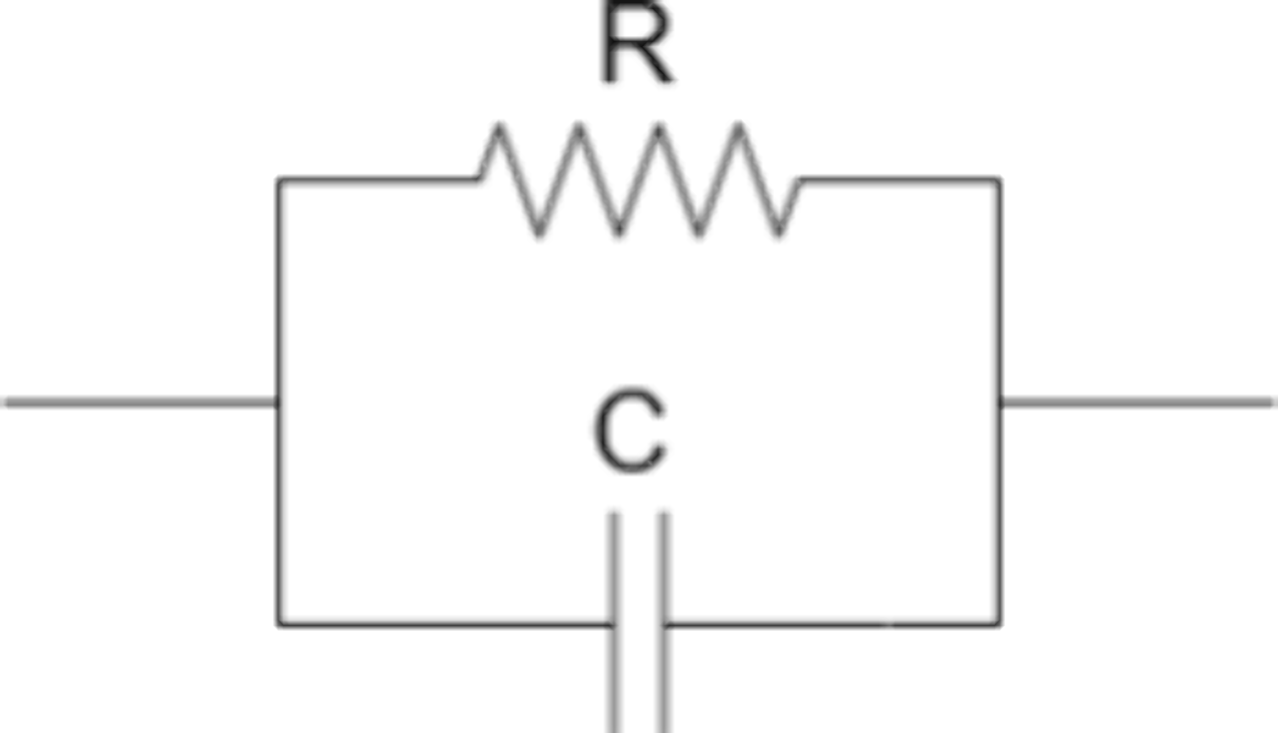
Equivalent circuit modeling of both fabricated sensors.

**Table 2:** Fitted Circuit Parameters at 60% RH for PBI and PY Sensors^a^

Parameter	PBI (mean ± std)	PY (mean ± std)
*R*^2^ (mag)	0.998 ± 0.001	0.997 ± 0.000
*R*^2^ (phase)	0.695 ± 0.327	0.982 ± 0.004
*R* (Ω)	4.32 × 10^8^ ± 4.06 × 10^8^	7.66 × 10^6^ ± 2.67 × 10^6^
*C* (F)	4.27 × 10^–12^ ± 4.11 × 10^–14^	4.99 × 10^–12^ ± 3.27 × 10^–13^

a Mean ± standard deviation.

The total impedance of the circuit is based on the following equation:

1 $$Z\left(\omega \right)={\left(\frac{1}{R}+\frac{1}{j\omega C}\right)}^{-1}$$

Lastly, a bending test was conducted on both sensors under ambient conditions, comparing their responses in a flat state and when curved to a radius of 1.5 mm (Figure S4). Upon bending, the impedance magnitude increased by 31.4% for the PBI sensor and 13.4% for the PY sensor. The phase angle changed by −1.3% for PBI and −14.7% for PY. This behavior is attributed to the mechanical deformation of the interdigitated electrode structure: as the fingers are elongated during bending, their separation increases, reducing capacitance (and thereby the phase), while the lengthened conductive path increases resistance. These mechanical effects do not directly alter the chemical sensing mechanism but introduce physical variations in the impedance signal that should be considered for real world applications. Although further investigation is needed to fully characterize these effects, such analysis falls outside the scope of this study.

### Response toward Temperature

Figure 12 illustrates the variation in phase and magnitude when the sensors are subjected to different temperatures at a constant 55% RH. For the PBI based sensor, the impedance response is essentially temperature independent, the phase exhibits no frequency-dependent shift, and the magnitude remains nearly constant. This behavior suggests that the PBI sensor is largely unaffected by temperature variations, likely owing to its mechanically and chemically stable backbone.^23,36,37^ Although the methoxy groups in PBI can act as hydrogen-bond acceptors, they interact only weakly with water, leading to a minimal temperature sensitivity.

**Figure 12:**
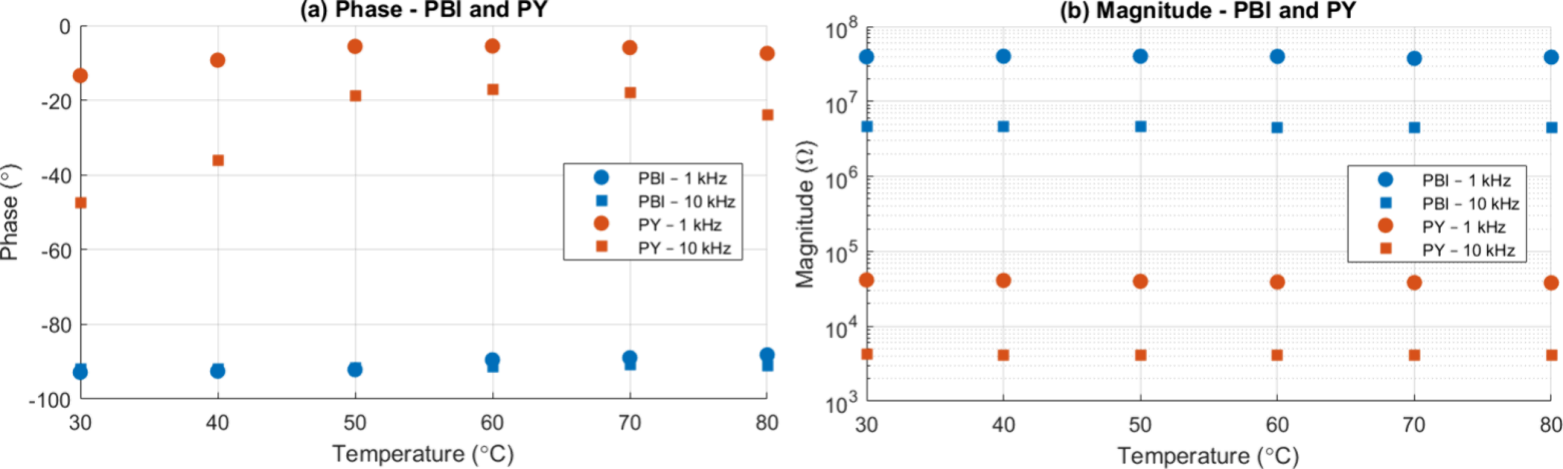
Impedance response to temperature at 55% RH for the developed sensors: (a) Phase angle and (b) impedance magnitude measured for PBI (blue) and PY (orange) devices while cycling the temperature from 30 to 80 °C. Circles and squares denote 1 kHz and 10 kHz read-out frequencies, respectively.

In contrast, the PY based sensor shows a modest, frequency-dependent temperature effect. Small changes in both phase and magnitude appear as temperature varies, reflecting the higher hydrophilicity of PY, its carboxylic-acid groups form stronger hydrogen bonds with water, making the impedance more susceptible to the temperature-driven redistribution of adsorbed moisture.^38,39^ While this temperature dependence is limited, a slight compensation may be required for precision humidity monitoring with PY devices.

To further assess thermal stability, additional measurements were performed at 70% RH and three temperatures (30, 50, and 80 °C). The results, presented in Table S1, show the phase and magnitude responses of the PBI and PY sensors, respectively. Although the absolute signal level decreases at higher temperatures, humidity sensitivity is preserved: the phase shift per % RH remains essentially unchanged. This demonstrates that the sensors retain functionality at elevated temperatures. Furthermore, the trend is monotonic and fully reversible across the tested range, which supports our claim of thermal robustness.

To evaluate the proposed humidity sensors relative to state-of-the-art devices, we compiled a benchmark comparison, presented in Table 3. The table contrasts our impedimetric sensors with representative humidity sensors that use resistive, capacitive, impedimetric, or optical read-out schemes. Our PY coating layer exhibits the steepest impedance slope among the printed impedimetric sensors surveyed, reaching −5.29 × 10^3^ Ω/% RH over the 30–90% RH window at 1 kHz. This value is roughly 1 order of magnitude larger than that of laser-induced-graphene/cork (1.5 × 10^3^ Ω/% RH) and three orders above the PBI control film prepared in this work (−4.52 × 10^2^ Ω/% RH). Relative to earlier perylene based architectures, viologen-PDI nanohelices, and K-Pery MOFs, the PY sensor delivers comparable or superior sensitivity while avoiding high-vacuum synthesis steps and retaining compatibility with flexible substrates. Thermal endurance is likewise competitive: both PY and PBI devices remain within specification from 30 to 80 °C, exceeding the 25–60 °C stability window reported for SnO_2_ nanostructures and matching the ≤10% drift observed for GO/PEDOT:PSS composites up to 60 °C. Consequently, no external temperature compensation circuitry is required for most ambient or wearable applications. A trade-off arises in transient behavior. At 30 Ω/% RH–90 Ω/% RH, the PY film requires ∼120 s for both response and recovery, whereas the thinner PBI analogue balances a lower sensitivity with 15 s/20 s transients. For ultrafast applications sub second breath monitoring, for example—capacitive GO/LIG interdigital electrodes or PVA/GQD fibers (1 s) still outperform our design. Nevertheless, the combination of high slope, broad thermal operating window, and simple printing confers a clear advantage for low power, distributed IoT nodes where sensitivity and fabrication simplicity are preferred over response time.

**Table 3:** Benchmark of This Work against Selected State-of-the-Art Humidity Sensors

Material (ref)	Sensor type	Test freq	Sensitivity^a^	Resp./Recov. (s)	Temp. stability
Viologen–PDI nanohelices^24^	Resistive	DC	2.9 × 10^5^ Ω/% (23–100)	21/29	N/R
K–Pery MOF^40^	Impedimetric	100 Hz	∼10^5^ decade drop @ 40%RH	N/R	N/R
Silica-gel perylene dye^41^	Optical	—	95% Δ*I* (0–100)	N/R	N/R
GO/PEDOT:PSS^42^	Capacitive	1 kHz	1.22 nF/% (30–75)	610/398	≤10% (25–60 °C)
GO + LIG IDEs^43^	Capacitive	1 kHz	1.8 nF/% (0–97)	16/9	Hysteresis 3.03 % (Stable cycling)
SnO_2_ nanostructures^44^	Resistive	DC	∼77 MΩ/% (11–96)	32/42	<5% hysteresis (25–60 °C)
Porous TiO_2_/polymer^45^	Capacitive	1 kHz	0.85 pF/% (10–90)	<35/N/R	Hysteresis 0.95 %;≤ 0.17% RH drift, 10–50 °C)
PVA/GQD hollow-core fiber^46^	Optical	—	117 pm/% (13–81)	0.9/1.2	N/R
LIG/Cork^47^	Resistive	500 Hz	1.5 kΩ/% (40–80)	140/77	Good (20–60 °C)
PY (This work)	Impedimetric	500 Hz–10 MHz	–5289 Ω @1 kHz (30–90)	120/120	Good (30–80 °C)
PBI (This work)	Impedimetric	500 Hz–10 MHz	–452 Ω @1 kHz (30–90)	15/20	Good (30–80 °C)

a Sensitivity as defined in each source (capacitance, resistance, or optical signal change per unit RH). N/R = not reported explicitly.

## Conclusion

In this study, we have explored the potential of functionalized perylene coated dispense printed electrodes for enhancing humidity sensing, presenting a comparative analysis between two sensor configurations: PBI based and PY based sensors. The PBI based sensors demonstrated a stable impedance response with minimal temperature dependence, making them suitable for applications where consistent performance is required under varying temperature conditions. In contrast, the PY based sensors exhibited a higher sensitivity toward RH and frequency dependent impedance with also minimal response to changes in temperature and enhanced sensitivity to humidity variations. This study highlights the role of chemical structure and functionalization in determining the sensor’s sensitivity and stability. The carboxylic acid groups in PY provided stronger hydrophilic interactions, which resulted in increased sensitivity to both humidity and temperature fluctuations, while the methoxy groups in PBI offered a more stable and temperature-insensitive response. Overall, this comparative study demonstrates that functionalized perylene coatings can significantly enhance the performance of flexible printed electrodes for humidity sensing. The insights gained from this work offer valuable guidance for the development of robust and efficient humidity sensors with applications spanning from environmental monitoring to precision agriculture and wearable technology.

## Supplementary Material



## Notes

Figures S1–S3: device photographs, ^1^H NMR spectrum, microscope images. Figures S4–S5: electrochemical impedance spectroscopy response of three devices toward relative humidity, mean ± SD. Figure S6: electrochemical impedance spectroscopy response of three devices toward temperature. Table S1: Additional temperature test. (PDF)
