# Supplementary material for: Enhancing Humidity Sensing with Functionalized Perylene-Coated Dispense Printed Electrodes: A Comparative Study
Source: ACS Appl Electron Mater. 2025 Jun 27;7(14):6311–21. doi: 10.1021/acsaelm.5c00360 (PMC12309082; doi:10.1021/acsaelm.5c00360)
Supplement: Supplementary file 1 [file el5c00360_si_001.pdf]

Supporting Information

Enhancing Humidity Sensing with Functionalized Perylene-Coated Dispense Printed Electrodes: A Comparative Study

Sahira Vasquez[1] , Samuel Morales Cámara [2] , Carmen Moraila[5] , Yann Houeix[5], Isabel Blasco Pascual[3], José F. Salmerón [5], Antonio Rodríguez-Diéguez[2], Sara Rojas [2] * , Niko Münzenrieder[4] , Luisa Petti[1*], Paolo Lugli [1] and Almudena Rivadeneyra [5*]

[1]Sensing Technologies Laboratory (STL), Faculty of Engineering, Free University of Bolzano-Bozen, via Bruno Buozzi 1, 39100 Bolzano, Italy

[2]Department of Inorganic Chemistry, Faculty of Science, University of Granada, 18071 Granada, Spain

[3]Department of Analytical Chemistry, University of Granada, 18071 Granada, Spain

[4]Faculty of Engineering, Free University of Bolzano-Bozen, via Bruno Buozzi 1, 39100 Bolzano, Italy

[5]Department Electronics and Computer Technology, University of Granada, 18071 Granada, Spain

*luisa.petti@unibz.it

(a) (b)

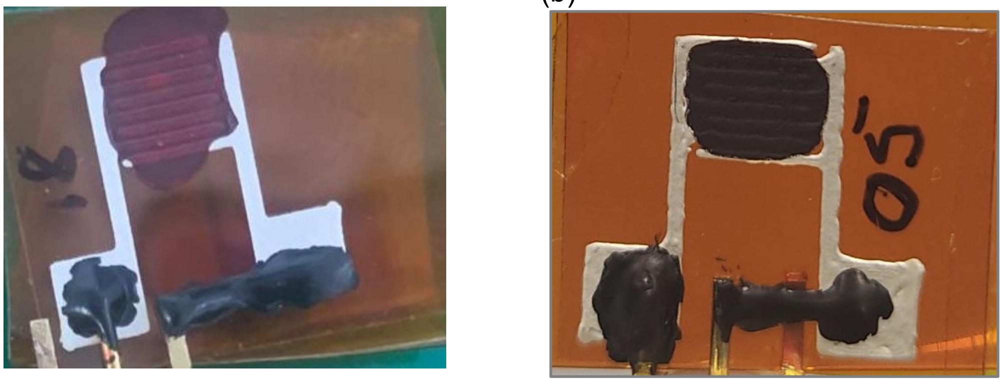

Figure S1. Images of the as-fabricated devices (a) PY and (b) PBI.

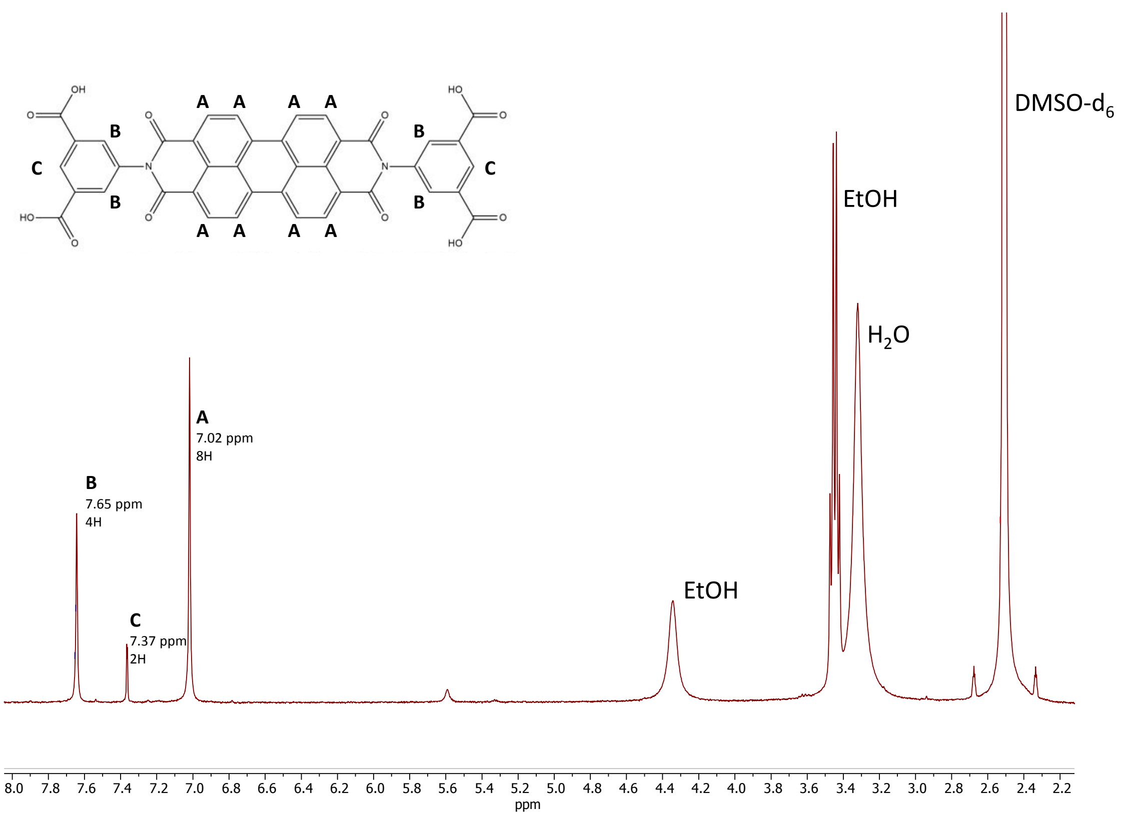

Figure S2. The ^{1}H-NMR (300 MHz, DMSO-d6) spectrum provided strong evidence to prove that PDI- PY ligand was synthesized successfully.

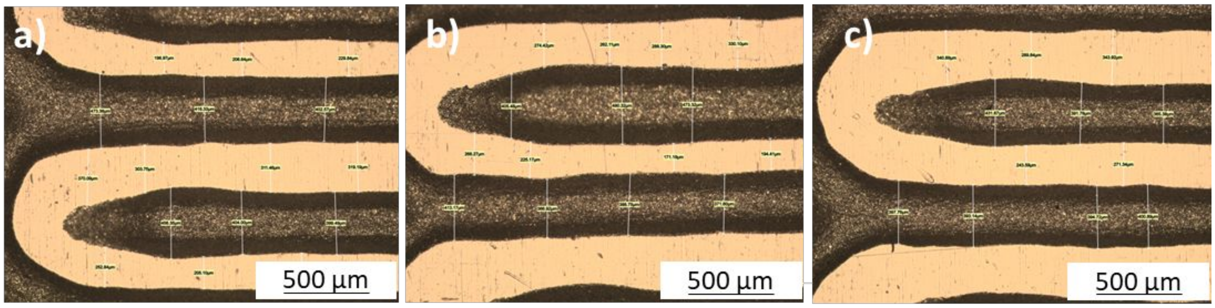

Figure S3. Optical microscope images of the printed silver IDEs. Panels (a–c) show representative regions of the IDEs with overlaid measurements indicating electrode width and gap spacing. The printed features show a consistent increase in line width (by 22%) and decrease in spacing (by 13%) relative to the design layout, consistent with prior dispense printing behavior.

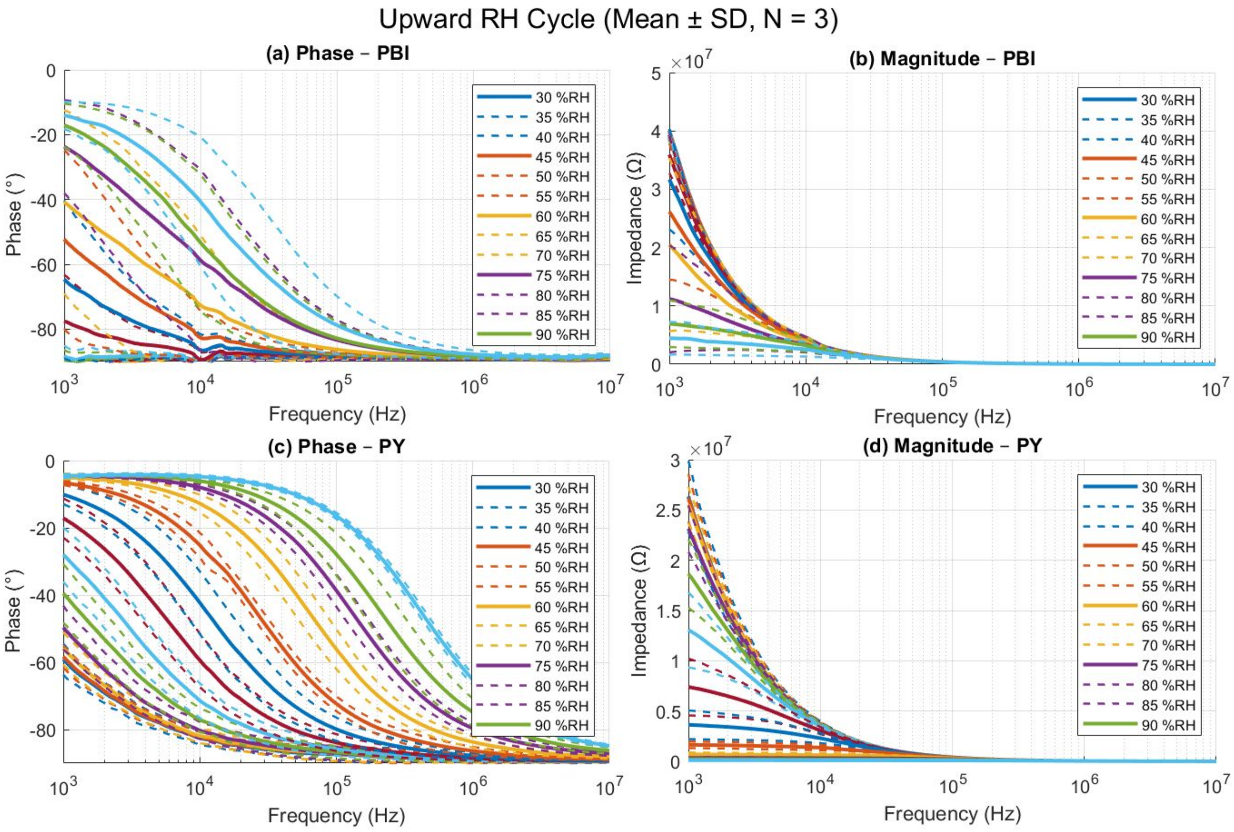

Figure S4. Electrochemical impedance spectroscopy (EIS) results for printed PBI and PY-based humidity sensors across a frequency range of 1 kHz to 10 MHz and relative humidity (RH) levels from 30% to 90% at 30°C. (a, b) Phase and magnitude responses of the PBI sensor. (c, d) Phase and magnitude responses of the PY sensor. Solid lines represent the mean values across three replicates; dashed lines indicate the corresponding standard deviation.

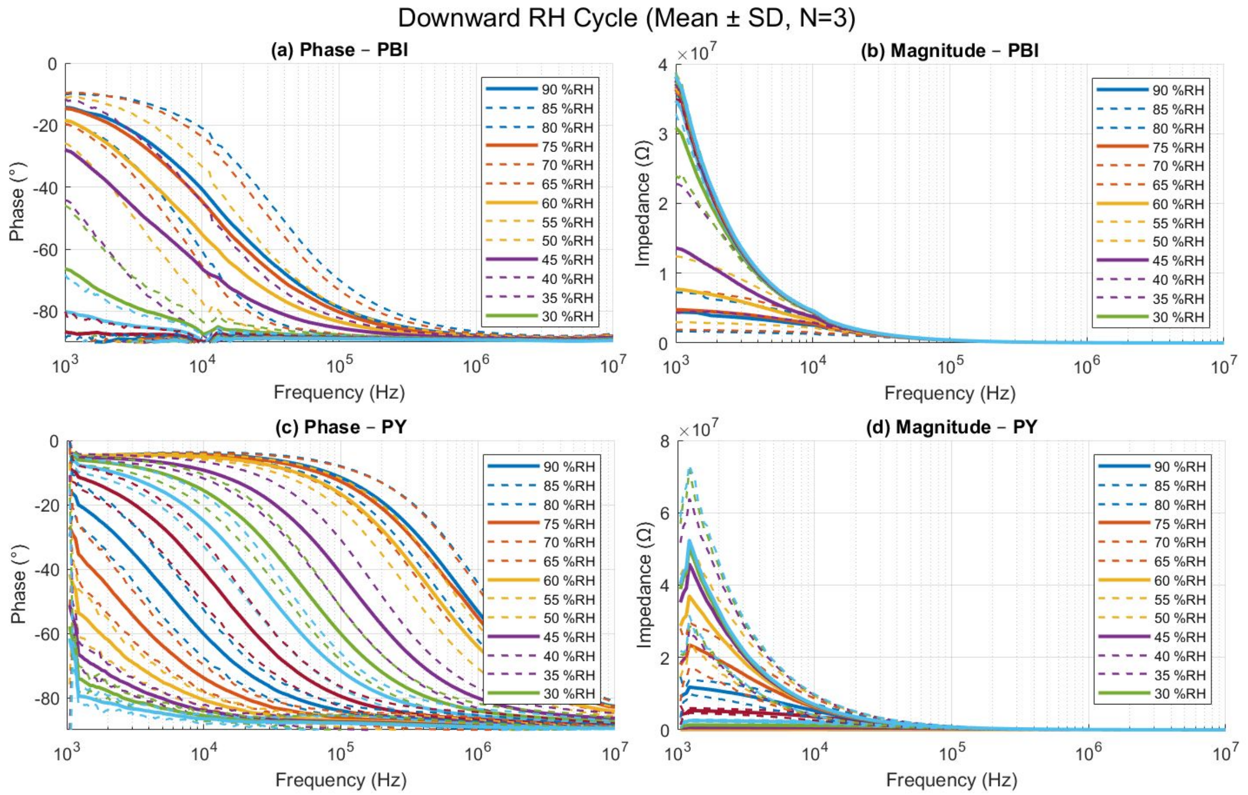

Figure S5. Electrochemical impedance spectroscopy (EIS) results for printed PBI and PY-based humidity sensors across a frequency range of 1 kHz to 10 MHz and relative humidity (RH) levels from 90% to 30% at 30°C. (a, b) Phase and magnitude responses of the PBI sensor. (c, d) Phase and magnitude responses of the PY sensor. Solid lines represent the mean values across three replicates; dashed lines indicate the corresponding standard deviation.

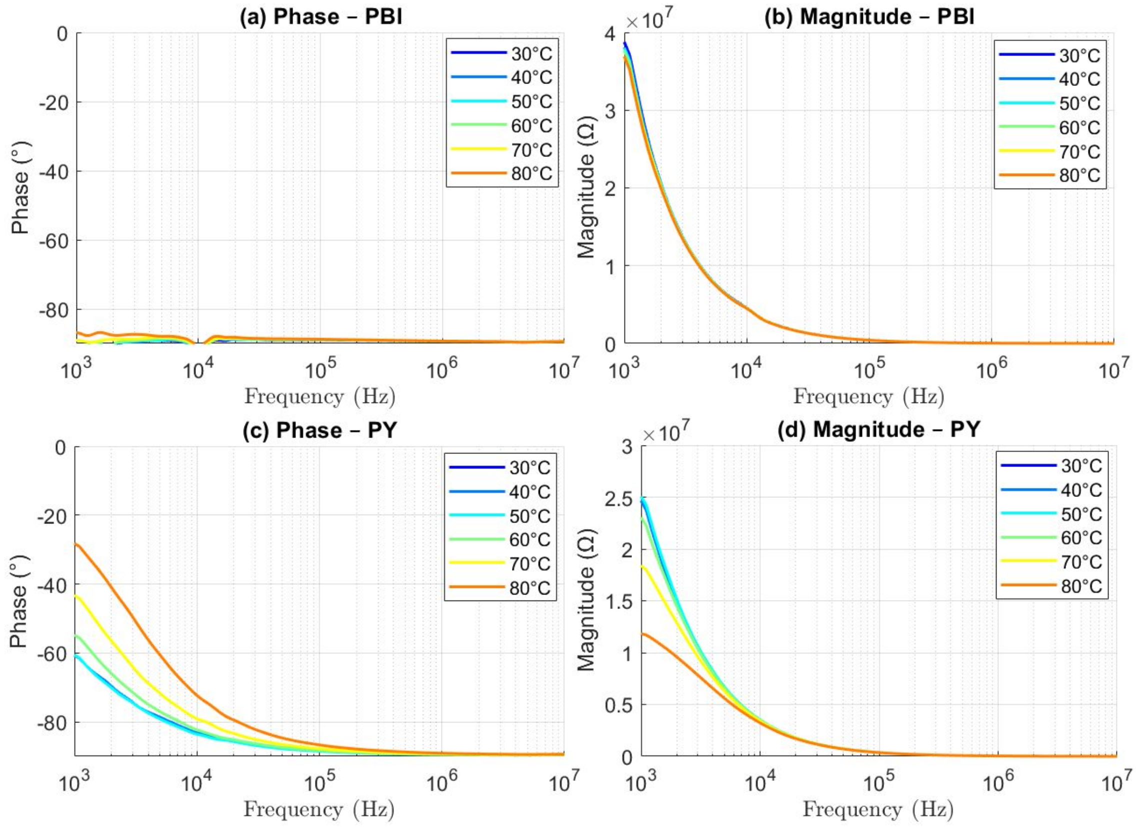

Figure S6. Electrochemical impedance spectroscopy (EIS) results for printed PBI and PY-based humidity sensors across a frequency range of 1 kHz to 10 MHz and temperature levels from 30°C to 80°C at 55%RH. (a, b) Phase and magnitude responses of the PBI sensor. (c, d) Phase and magnitude responses of the PY sensor.

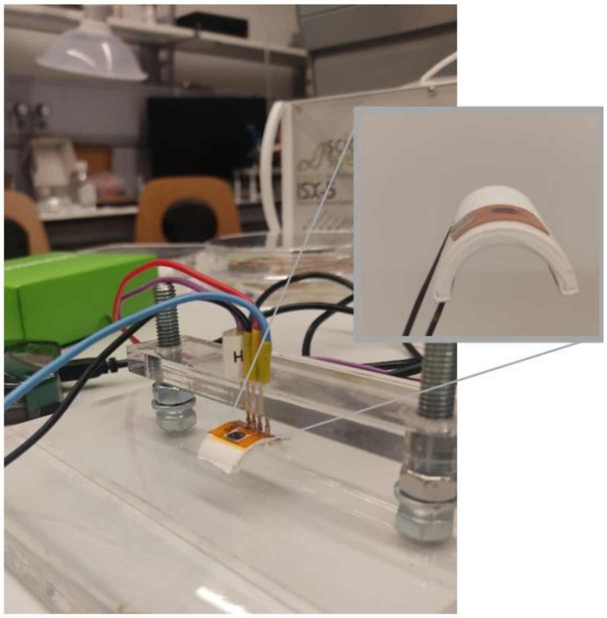

Figure S7 Photograph of the bending test setup used to evaluate the mechanical flexibility of the printed humidity sensor. The sensor was mounted on a custom-built rig allowing repeated bending at a fixed curvature radius. The inset shows a close-up of the sensor under mechanical deformation, demonstrating its conformability to curved surfaces.

Table S1. Phase and magnitude response of PBI and PY sensors at 1 kHz and 70% RH under different temperatures.

Temperature (°C)	PBI Phase (°)	PBI Magnitude (Ω)	PY Phase (°)	PY Magnitude (Ω)
30	-77	69240500	-84	900005900
50	-69	59959400	-75	78935000
80	-64	29134800	-57	25532500
